# Adult age differences in frontostriatal representation of prediction error but not reward outcome

**DOI:** 10.3758/s13415-014-0297-4

**Published:** 2014-05-23

**Authors:** Gregory R. Samanez-Larkin, Darrell A. Worthy, Rui Mata, Samuel M. McClure, Brian Knutson

**Affiliations:** 1 Department of Psychology, Yale University, 2 Hillhouse Avenue, Box 208205, New Haven, CT 06520-8205 USA; 2 Department of Psychology, Texas A&M University, College Station, TX USA; 3 Department of Psychology, University of Basel, Basel, Switzerland; 4 Max Planck Institute for Human Development, Berlin, Germany; 5 Department of Psychology, Stanford University, Stanford, CA USA

**Keywords:** Aging, Reward, Motivation, Learning, Decision making, Medial prefrontal cortex, Ventral striatum

## Abstract

Emerging evidence from decision neuroscience suggests that although younger and older adults show similar frontostriatal representations of reward magnitude, older adults often show deficits in feedback-driven reinforcement learning. In the present study, healthy adults completed reward-based tasks that did or did not depend on probabilistic learning, while undergoing functional neuroimaging. We observed reductions in the frontostriatal representation of prediction errors during probabilistic learning in older adults. In contrast, we found evidence for stability across adulthood in the representation of reward outcome in a task that did not require learning. Together, the results identify changes across adulthood in the dynamic coding of relational representations of feedback, in spite of preserved reward sensitivity in old age. Overall, the results suggest that the neural representation of prediction error, but not reward outcome, is reduced in old age. These findings reveal a potential dissociation between cognition and motivation with age and identify a potential mechanism for explaining changes in learning-dependent decision making in old adulthood.

**Electronic supplementary material:**

The online version of this article (doi:10.3758/s13415-014-0297-4) contains supplementary material, which is available to authorized users.

Adult development and aging has been associated with relative stability in some aspects of motivational function but with decreases in some aspects of cognitive function (Carstensen, 2006; Grady, 2008; Reuter-Lorenz & Lustig, 2005; Samanez-Larkin & Carstensen, 2011; Samson & Barnes, 2013). Until recently, the majority of studies into the cognitive neuroscience of aging have examined separately either motivational or cognitive function. However, an emerging literature on age differences in reward learning and decision making has facilitated the examination of potential overlap or dissociation in these processes. The initial set of neuroimaging findings from this literature attributed deficits in reward learning in older age to structural and functional differences in frontostriatal circuitry (Eppinger, Hämmerer, & Li, 2011; Hämmerer & Eppinger, 2012; Samanez-Larkin & Knutson, 2014). Although older adults show intact, or even enhanced, frontostriatal responses to reward outcomes (Cox, Aizenstein, & Fiez, 2008; Samanez-Larkin et al., 2007; Schott et al., 2007), they also show decreased ventral striatal function in time-limited learning tasks (Chowdhury et al., 2013; Eppinger, Schuck, Nystrom, & Cohen, 2013; Mell et al., 2009; Samanez-Larkin, Kuhnen, Yoo, & Knutson, 2010). Some have suggested that adult age differences in reward-based decision making are due to a motivational deficit, such that older adults are less sensitive to reward than are younger adults (Eppinger, Nystrom, & Cohen, 2012). However, an alternative account of these collected findings may be that, whereas sensitivity to reward and previously learned reward associations remain intact over the adult lifespan, a network of neural systems that supports novel reward learning changes with age. This view suggests a potential dissociation between motivation and cognition in the aging brain.

Accordingly, a recent review of behavioral research showed the largest and most reliable adult age differences in decision tasks that depend on learning novel stimulus–reward associations, but few age differences in tasks that did not require recent learning (Mata, Josef, Samanez-Larkin, & Hertwig, 2011). Building on these findings, a diffusion tensor-imaging study revealed that the structural connectivity of the prefrontal cortex to the striatum could account for age differences in probabilistic reward learning (Samanez-Larkin, Levens, Perry, Dougherty, & Knutson, 2012). Together, this prior evidence has suggested that although older adults show intact sensitivity to reward magnitude (Samanez-Larkin et al., 2007), decision-making deficits in older age may result from decreased frontostriatal connectivity. This reduction in connectivity may compromise the dynamic updating of reward predictions (Eppinger et al., 2011).

A pair of recent studies have begun to determine which specific facets of learning-related brain activity are disrupted with age, by combining functional neuroimaging with models of reinforcement learning (Chowdhury et al., 2013; Eppinger et al., 2013). Reinforcement-learning models rely on the computation of prediction errors (i.e., the difference between the expected and received rewards), which are used to inform subsequent actions and maximize reward over time (O’Doherty, 2004; Pessiglione, Seymour, Flandin, Dolan, & Frith, 2006; Schönberg, Daw, Joel, & O’Doherty, 2007; Sutton & Barto, 1998). Neural representations of prediction error are believed to originate in the midbrain (Hollerman & Schultz, 1998; Schultz, 2006; Schultz, Dayan, & Montague, 1997) and have been observed in human neuroimaging across a range of midbrain, ventral striatal, and medial frontal regions in young adults (D’Ardenne, McClure, Nystrom, & Cohen, 2008; McClure, Berns, & Montague, 2003; O’Doherty, Dayan, Friston, Critchley, & Dolan, 2003; Pagnoni, Zink, Montague, & Berns, 2002); all of these regions are efferent targets of ascending dopamine projections of the mesolimbic path (Haber & Knutson, 2010; O’Doherty, 2004). Two recent studies have identified adult age-related reductions in the neural representation of prediction errors during learning in the ventral striatum and medial frontal cortex (Eppinger et al., 2013), and shown that dopaminergic drugs can enhance both learning ability and prediction error signals in the ventral striatum in old age.

Although these studies have made great progress in characterizing age differences in this reward-learning-related neural signal, one limitation of the current literature is that no prior studies have compared reward-feedback-related activation in tasks that do or do not depend on learning in the same subjects across adulthood. The goal of the present research was to compare the neural activity associated with prediction error across adult age, and further to determine whether age differences were limited to learning tasks or extended more generally to reward tasks that do not require learning. Accordingly, we conducted neuroimaging and behavioral studies in a community sample of healthy adults. In the first study, an adult lifespan sample of young, middle-aged, and older adults completed reward tasks that did or did not require learning, while undergoing functional magnetic resonance imaging (fMRI). In a second, behavioral study, we examined the behavioral consequences of age differences in prediction error coding by testing the limits of older adults’ learning ability through various task demand manipulations (e.g., choice set size and time available to learn) in a different sample of subjects.

## Study 1

### Method

#### Subjects

A group of 39 healthy, right-handed adults (mean age = 53 years, *SD* = 16, range 22–85; 21 female, 18 male) completed a probabilistic reward-learning task while undergoing fMRI. A subset of 37 adults (mean age = 52 years, *SD* = 16, range 22–85; 20 female, 17 male) also completed a reward task that did not depend on learning while undergoing fMRI (see Supplementary Information S1). After completing the reward tasks that either did or did not depend on learning, subjects also completed a risky decision-making task while undergoing fMRI. The results from the third task have appeared in a previous publication (Samanez-Larkin et al., 2010). A market research firm initially contacted individuals who were representative of San Francisco area residents with respect to sex, income, education, ethnicity, and occupation. Age was uniformly distributed across the sample, and all subjects had globally intact cognitive performance, as evidenced by Mini-Mental State Exam scores >26. All subjects gave written informed consent, and the experiment was approved by the Institutional Review Board of Stanford University.

#### Monetary incentive learning (MIL) task

To examine age differences in the functional representation of prediction error during probabilistic learning, all 39 subjects completed the MIL task (Knutson, Samanez-Larkin, & Kuhnen, 2011; Samanez-Larkin, Hollon, Carstensen, & Knutson, 2008; Samanez-Larkin et al., 2012) while undergoing fMRI. On each trial, subjects saw and chose between one of three pairs of fractal cues (gain acquisition, loss avoidance, or neutral). After choosing one of the cues from a pair, subjects saw the outcome associated with their choice (see Supplementary Information S2). On average, one of the cues yielded a better outcome, whereas the other yielded a worse outcome. In *gain cue* pairs, the better cue had a higher probability of returning gains (66 % +$1.00 returns and 33 % +$0.00 returns) than did the worse cue (33 % +1.00 and 66 % +$0.00); likewise, in loss cue pairs, the better cue had a higher probability of returning nonlosses (66 % –$0.00 and 33 % –$1.00) than did the worse cue (33 % –$0.00 and 66 % –$1.00). In neutral cue pairs, the choice of either cue had no impact on the outcomes (100 % $0.00). Each trial lasted 10 s, and intertrial intervals were drawn from a uniform distribution of 2, 4, or 6 s. The three trial types were each presented 24 times in an individually randomized order for each subject. Subjects completed a total of 72 trials. Within each cue pair, the cues appeared with equal frequency on the left or right side of the screen. The computer randomly assigned each cue to either the better or the worse outcome distribution at the beginning of each run, in a counterbalanced fashion. Different cue pairs were used for the practice and experimental sessions, in order to minimize memory-related interference. Subjects were explicitly informed about the cue probabilities before the practice session and told to try to maximize their earnings throughout the experiment. The subjects received cash for their performance after the experimental sessions, but not for the practice sessions. Measures of learning performance were assessed by calculating the percentages of choices that matched the “better” cue (i.e., had the higher probability of an advantageous outcome; Knutson et al., 2011; Samanez-Larkin et al., 2012).

#### Monetary incentive delay (MID) task

To examine the functional representation of reward outcomes in the absence of probabilistic learning, 37 subjects also completed the MID task (Knutson, Fong, Bennett, Adams, & Hommer, 2003; Samanez-Larkin et al., 2007; Wu, Samanez-Larkin, Katovich, & Knutson, 2014) while undergoing fMRI. On each trial, subjects saw a cue, responded with a buttonpress to a target, and then received feedback (see Supplemental Information S2). Each trial lasted 8 s, and intertrial intervals were drawn from a uniform distribution of 2, 4, or 6 s. A total of six cue types (Win $0.00, Win $0.50, Win $5.00, Lose $0.00, Lose $0.50, and Lose $5.00) explicitly stated whether each trial was a potential gain or loss trial, as well as the amount of money at stake. The six trial types were each presented 15 times in an individually randomized order for each subject. The subjects completed a total of 90 trials. Task performance was manipulated by altering the average duration of the target with an adaptive timing algorithm (seeded with that individual’s mean reaction time in prescan practice) that tracked performance across the task to maintain a 66 % hit rate for each cue type.

#### fMRI data collection and analysis

Neuroimaging data were collected using a 1.5-T General Electric MRI scanner using a standard birdcage quadrature head coil. High-resolution structural scans were acquired using a T1-weighted spoiled GRASS sequence (TR = 100 ms, TE = 7 ms, flip = 90º), facilitating localization and coregistration of the functional data. After acquiring the anatomical scans, all subjects first completed the MID task and then the MIL task. Twenty-four 4-mm-thick slices (in-plane resolution 3.75 × 3.75 mm, no gap) extended axially from the mid-pons to the top of the skull. Functional scans of the whole brain were acquired at a repetition time of 2 s, with a T2*-sensitive in-/out- spiral pulse sequence (TE = 40 ms, flip = 90º) designed to minimize signal dropout at the base of the brain (Glover & Law, 2001). Preprocessing and whole-brain analyses were conducted using the AFNI (Analysis of Functional Neural Images) software (Cox, 1996). For preprocessing, voxel time series were sync-interpolated to correct for nonsimultaneous slice acquisition within each volume, corrected for three-dimensional motion, slightly spatially smoothed (FWHM = 4 mm), converted to percentage signal change (relative to the mean activation over the entire experiment), and high-pass filtered. Visual inspection of the motion correction estimates confirmed that no subject’s head moved more than 4 mm in any dimension from one volume acquisition to the next.

The preprocessed time series data for each individual were used in two sets of analyses. A first set of time-course-based analyses examined age differences in select brain regions, based on recent evidence for abnormal prediction error signaling and structural declines in the medial frontal cortex and ventral striatum in old age (Chowdhury et al., 2013; Eppinger et al., 2013; Samanez-Larkin et al., 2012). For these time-course-based analyses, volumes of interest were specified anatomically on the basis of previous studies of age differences in learning and decision making (Samanez-Larkin et al., 2010; Samanez-Larkin et al., 2012), and measures of percentage signal change were extracted from the same regions by conditions of interest in both the MIL and MID tasks. These 8-mm-diameter spheres were shifted within individuals to ensure that only data from gray matter were extracted.

A second set of analyses used multiple regression to examine group effects and age differences across the whole brain (see Supplementary Information S3). In the whole-brain analyses, the regressors of interest were convolved with a gamma-variate function that modeled a prototypical hemodynamic response before inclusion in the regression model.

The MIL task whole-brain regression model consisted of a set of two orthogonal regressors of interest: prediction error on gain trials, and prediction error on loss trials. In this parametric model, the prediction errors were fully signed and varied across trials and subjects. For full details on the estimation of prediction error and model fit, see Supplementary Information S3. Additional regressors of noninterest included residual motion and baseline, linear, and quadratic trends.

The MID task regression model consisted of a set of four orthogonal regressors of interest: gain ($0.50, $5.00) versus nongain ($0.00) anticipation, loss ($0.50, $5.00) versus nonloss ($0.00) anticipation, gain (hit: $0.50, $5.00) versus nongain (miss: $0.50, $5.00) outcome, and nonloss (hit: $0.50, $5.00) versus loss (miss: $0.50, $5.00) outcome. Additional regressors of noninterest included task periods (anticipation and outcome), residual motion, and baseline, linear, and quadratic trends.

Maps of the *t* statistics representing each of the regressors of interest were transformed into *z* scores, resampled at 3.75 mm^3^, and spatially normalized by warping to Talairach space. These *β* coefficient maps were then regressed on linear and quadratic age effects (continuous independent variables). The independent variables were mean-centered so that the resulting model intercept revealed regions of the brain that correlated significantly with that regressor of interest across the sample, controlling for age. Voxelwise thresholds for statistical significance at the whole-brain level were set at *p* < .001, uncorrected. The minimum cluster size of seven 3.75-mm^3^ voxels for a *p* < .05 whole-brain corrected threshold was estimated using AFNI’s AlphaSim (Cox, 1996). Small-volume correction was applied to the ventral striatum at the same threshold (*p* < .001) by removing the cluster criterion (which was too large to allow for detection of activation in regions as small as the nucleus accumbens).

In all fMRI analyses, care was taken to minimize potential confounds associated with age differences in subject characteristics, brain morphology, and hemodynamics (Samanez-Larkin & D’Esposito, 2008). What appear to be main effects of age reported in the tables are analogous to Age × Condition interactions, since the dependent variables in these models are coefficient maps resulting from the first-level analyses. Each individual’s brain was warped into Talairach space with reference to hand-placed anatomical landmarks. The structural and functional brain-imaging data were inspected for abnormalities in each individual. Four additional individuals not included in the numbers reported above were excluded from all analyses, because of a structural abnormality (71-year-old male), excessive motion (26-year-old male, 74-year-old male), or extreme BOLD signal change values (>3 *SD*s above/below the sample mean for contrasts of interest; 25-year-old male).

Although gain and loss conditions were included in both tasks, all results and discussion focused on MIL gain learning and MID gain outcome trials. Prior research had revealed age differences in the processing of monetary losses, even in the absence of learning (Samanez-Larkin et al., 2007; Wu et al., 2014). The goal of the present study, however, was to examine how age differences in gain learning emerged, given prior evidence for the preservation of reward magnitude representations in old age. Full results from the loss conditions appear in Supplementary Information S6.

### Results

To examine age differences in neural responses during reward learning (even in the face of preserved responses to reward outcomes), 39 healthy adults of varying ages (age range 22–85) completed a probabilistic-learning task while undergoing fMRI. Given prior evidence for age differences in the processing of monetary losses, even in the absence of learning (Samanez-Larkin et al., 2007; Wu et al., 2014), our analyses focused on the gain-learning conditions (findings from the loss conditions appear in Supplementary Information S6).

#### MIL task behavioral results

The results with a larger sample size (*N* = 77) showed age differences (main effect of age) in performance on the MIL task. This finding results from the fact that older adults less often chose the higher expected value cue during both gain and loss learning—particularly during the early phase of learning (see Supplementary Information S4). The Age × Valence (gain, loss) interaction was not significant in this larger sample (see Supplementary Information S4), suggesting that older subjects learned less from probabilistic feedback overall. In the subsample of subjects who underwent fMRI (*N* = 39), averaged performance across both gain and loss learning was not associated with age, *β* = –.26, *p* = .10, possibly due to a lack of power to detect behavioral effects in this smaller subsample.

#### MIL task neural results

**Fig. 1:**
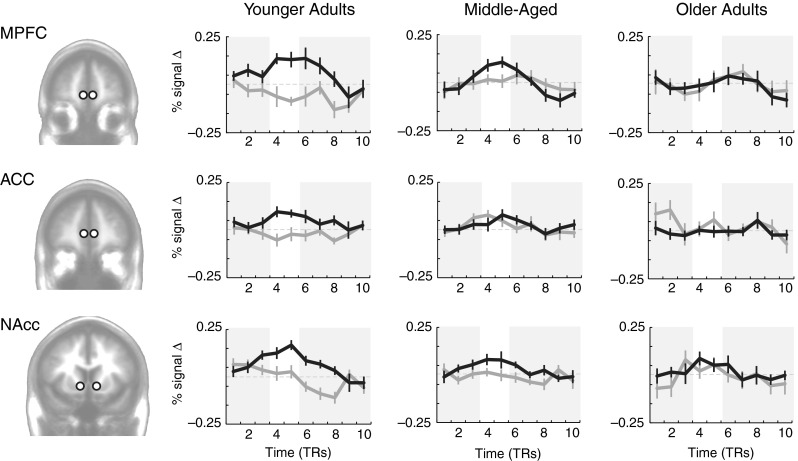
Reward-learning (MIL) task, time-course-based results: Time courses of activation comparing positive to negative prediction errors during the learning task in younger (age range 22–44; *N* = 12), middle-aged (age range 45–60; *N* = 13), and older (age range 64–85; *N* = 14) adults. Black lines are positive prediction errors, and gray lines are negative prediction errors. MPFC = medial prefrontal cortex; ACC = anterior cingulate cortex; NAcc = nucleus accumbens. Error bars indicate *SEM*s. White regions highlight feedback intervals adjusted for hemodynamic lag

In initial analyses, we examined activation time courses extracted from the bilateral medial prefrontal cortex (MPFC), anterior cingulate cortex (ACC), and nucleus accumbens (NAcc) (Fig. 1), on the basis of recently reported learning-related age differences in frontal cortex and striatum (Eppinger et al., 2013; Samanez-Larkin et al., 2012). Beyond the regions of interest we focused on here, a larger circuit including the midbrain, striatum, and prefrontal cortex is involved in the computation and representation of values and prediction errors. Although some have suggested that age differences may primarily be due to rising dopamine from the midbrain to the ventral striatum and MPFC (e.g., Chowdhury et al., 2013, Eppinger et al., 2013), other evidence suggests that these effects may be more distributed and may be broader than a purely dopaminergic decline with age (Samanez-Larkin et al., 2012). These prior studies together informed our selection of regions in the present article.

Within these brain regions of interest, positive prediction error time courses were constructed from the average of the signals from all trials with positive prediction errors. Similarly, negative prediction error time courses were constructed from the average of signals from all trials with negative prediction errors. Trials were included in these averages on the basis of prediction errors estimated from the model described in the supplementary information (S3). Across age groups, activation was significantly higher on trials with a positive than with a negative prediction error in the MPFC, *t*(38) = 3.40, *p* < .001, and NAcc, *t*(38) = 3.12, *p* < .01, but not in the ACC, *t*(38) = 0.85, *p* = .39. The difference in neural activity between trials in which we found a positive versus a negative prediction error decreased as age increased in the MPFC, *β* = –.45, *p* < .01, NAcc, *β* = –.39, *p* < .05, and the ACC, *β* = –.46, *p* < .01.

**Fig. 2:**
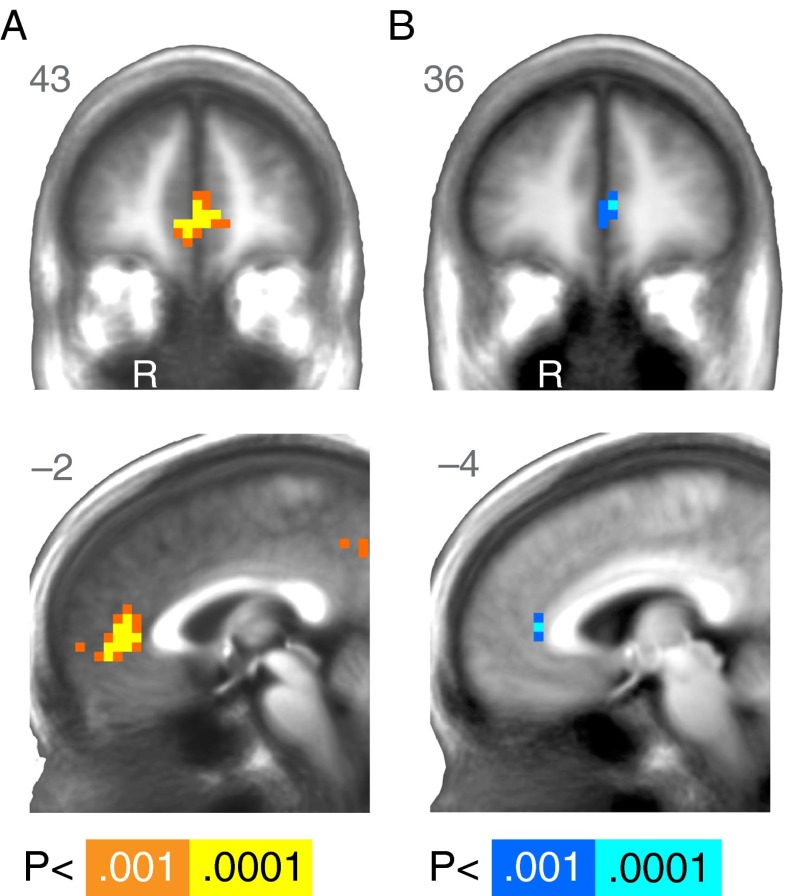
Reward-learning (MIL) task, whole-brain results. (A) Regions of the brain where activation was significantly modulated by prediction error at outcome across age during learning. (B) Regions of the brain where the modulation of activation by prediction error showed age differences. Cooler colors correspond to negative *z* scores, which indicate a reduced modulation of activation as age increased. R = right. A right/left, anterior/posterior, or superior/inferior value is listed in the upper corner of each statistical map. The anatomical underlay is an average of all subjects’ spatially normalized structural scans

For whole-brain analyses, we used a reinforcement-learning model to fit behavior and generate estimates of prediction error at each trial (see Supplementary Information S3). We then used estimated prediction errors to identify brain areas that correlated with this signal during learning. Unlike the time course analyses above, which collapsed all prediction errors independent of size, the whole-brain analyses provided coefficients for the parametric effect of prediction error. The whole-brain analysis identified a cluster in the MPFC in which activation correlated with prediction error across age groups (Table 1, Fig. 2A). Differences as a function of age were evident in the correlation between neural activity and prediction errors. At a whole-brain cluster-corrected threshold, age differences emerged in ACC activation, indicating a greater correlation for younger than for older subjects (Table 1, Fig. 2B) consistent with the time-course-based analyses above.

**Table 1:** Regions modulated by prediction error at outcome in the task that required learning (MIL)

Region	R	A	S	Z	Voxels	Volume
Across All Subjects						
R Medial frontal gyrus	4	49	–3	5.17	66	3,480
R Ventral putamen / Nucleus accumbens	15	8	–7	3.87	4 [SVC]	211
R Posterior cingulate gyrus	8	–49	34	4.27	18	949
L Inferior parietal lobule	–52	–49	38	3.95	8	422
Age Differences (Older > Younger)						
L Anterior cingulate	–4	38	8	–3.98	7	369
R Middle temporal gyrus	49	–34	1	–4.02	9	475
L Posterior cingulate	–11	–41	4	–3.65	9	475
Quadratic Effect of Age						
none						

Average effects are reported across all subjects, along with main and quadratic effects of age. *p* < .001 for all activations, *N* = 39. SVC, small volume corrected

#### MID task neural results

**Fig. 3:**
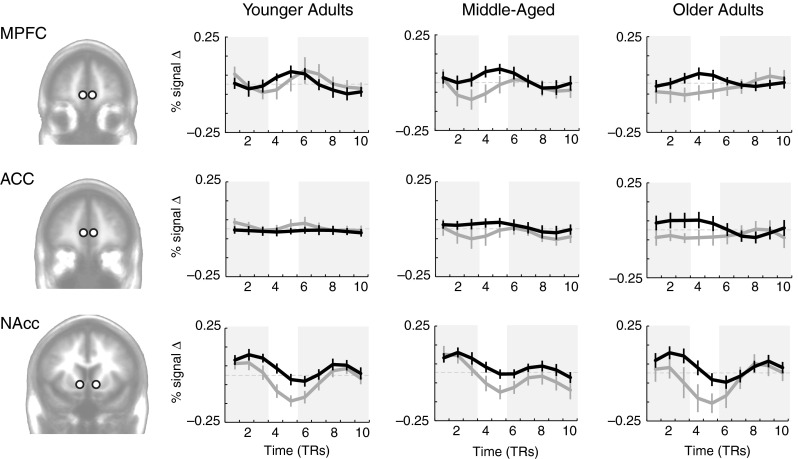
Simple-reward (MID) task, time-course-based results: Time courses of activation comparing gains to nongains during the task that did not require learning in younger (age range 22–44; *N* = 12), middle-aged (age range 45–60; *N* = 13), and older (age range 64–85; *N* = 12) adults. Black lines are gain outcomes (+$0.50, +$5.00), and gray lines are nongain (+$0) outcomes. MPFC = medial prefrontal cortex; ACC = anterior cingulate cortex; NAcc = nucleus accumbens. Error bars indicate *SEM*s. White regions highlight feedback intervals adjusted for hemodynamic lag

A subset of 37 of the adults (age range 22–85 years) also completed the MID task while undergoing fMRI. Unlike the MIL task, performance on the MID task does not require learning. As above, we began our analysis with time courses extracted from predicted volumes of interest and the followed these analyses with whole-brain regression. Across age groups, activity was significantly higher for gain than for nongain outcomes in the MPFC, *t*(36) = 3.57, *p* < .01, and NAcc, *t*(36) = 5.82, *p* < .0001, and marginally higher in the ACC, *t*(36) = 1.93, *p* = .06. Furthermore, the difference in activity between gain and nongain outcomes did not vary across age groups in the MPFC, *β* = .18, *p* = .29, or NAcc, *β* = .11, *p* = .53 (Fig. 3). In fact, unlike the learning task (MIL), the difference between gain and nongain outcomes in the MID task increased with age in the ACC, *β* = .40, *p* < .05. The findings replicated prior evidence for preserved frontostriatal functional reward activation in tasks that do not require learning.

**Fig. 4:**
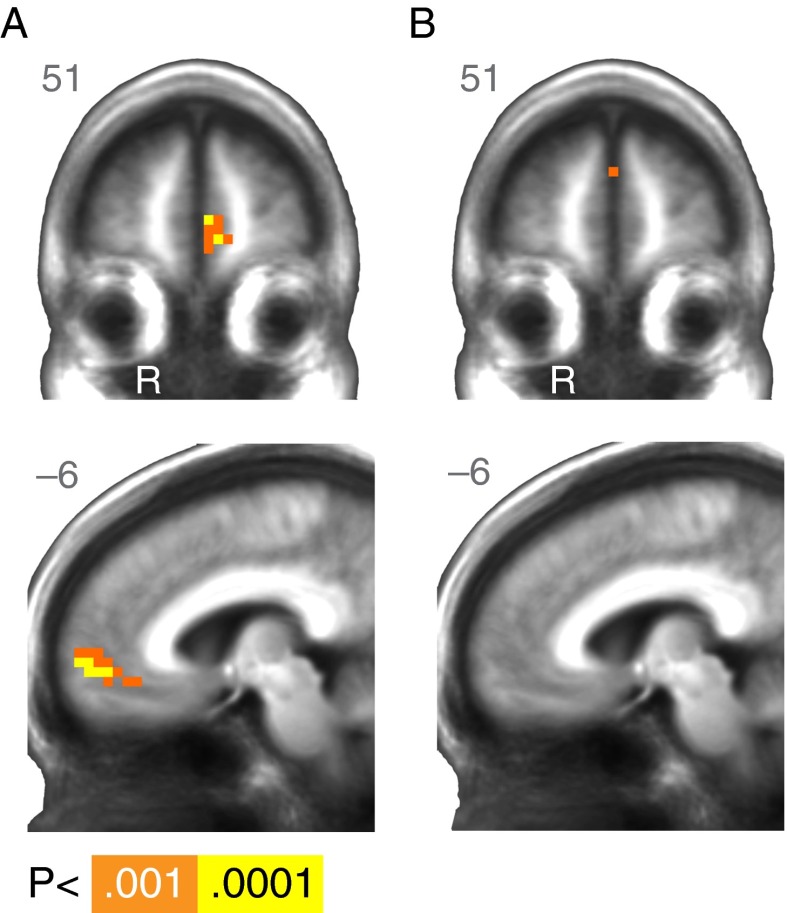
Simple-reward (MID) task, whole-brain results. (A) Regions of the brain where activation was significantly greater for monetary gains than for nongains at outcome across age. (B) No significant age differences in reward modulation were apparent at outcome in the MID task. R = right. A right/left, anterior/posterior, or superior/inferior value is listed in the upper corner of each statistical map. The anatomical underlay is an average of all subjects’ spatially normalized structural scans

The whole-brain analysis of reward outcome in the MID task revealed a cluster in the medial frontal cortex (Table 2, Fig. 4A) where activation was modulated by reward outcome (gain vs. nongain), aggregating across all subjects. At the whole-brain cluster-corrected threshold, no regions showed significant effects of age (Table 2, Fig. 4B).

**Table 2:** Regions modulated by reward outcome (+$ vs. +0) in the task that did not require learning (MID)

Region	R	A	S	Z	Voxels	Volume (mm^3^)
Across All Subjects						
L Medial frontal gyrus	–11	60	1	4.24	53	2,795
L Middle frontal gyrus	–22	4	46	4.01	8	422
Linear Effect of Age (OA > YA)						
none						
Quadratic Effect of Age						
R Inferior frontal gyrus	30	11	–14	–4.89	10	527
L Inferior frontal gyrus	–30	8	–11	3.81	10	527
R Lentiform nucleus / Amygdala	23	0	–11	3.69	7	369
R Precuneus	4	–79	42	–3.88	19	1,002
R Cuneus	11	–90	4	–4.99	31	1,635

Average effects are reported across all subjects, along with main and quadratic effects of age. *p* < .001 for all activations, *N* = 37

### Interim discussion

On the basis of the evidence that neural activity associated with prediction errors is reduced at older ages in Study 1, particularly in the prefrontal cortex, we predicted that age differences in learning would be magnified as learning demands increased. To test this hypothesis, a separate group of 18 younger adult (age range 19–33) and 30 older adult (age range 67–86) subjects completed a behavioral task that included twice as many cues as the MIL task (four instead of two) from which to choose and learn. Additionally, we wanted to test the hypothesis that even in this more demanding learning task, older adults could perform as well as younger adults if given adequate time. Specifically, if deficits exist in learning from feedback (as indicated by the results above), performance should differ by age during early stages of learning, but not at asymptote. By contrast, were age-related differences to exist in the reward signals themselves (which was not suggested by the neuroimaging data in Study 1), then learning should be biased in a manner than could not be overcome with more learning trials.

## Study 2

### Method

#### Subjects

Eighteen younger (ages 19–33) and 30 older (ages 67–86) adults completed a modified version of the MIL task on a laptop in an interview room in the Psychology Department (see Supplementary Information S1). Study 2 did not include fMRI. Right-handed subjects were recruited either by a market research firm or through local online advertisements (e.g., Craigslist) in the San Francisco Bay area.

#### Expanded MIL task

On each trial, subjects saw and chose between fractal cues and then viewed the outcome associated with their choices. The expanded MIL task was different from the standard MIL task described above in Study 1, in four ways. First, the task used in Study 2 only included a gain condition (without a loss condition). Second, the choice set size was doubled, so that subjects chose from four (instead of two) cues on each trial. The probabilities of winning $1 associated with each of the four cues were 40 %, 50 %, 60 %, and 70 %. Third, in this task subjects were not given the probability distributions as priors. The only information provided was that in each round some cues would be better than others (i.e., would be associated with a higher probability of winning $1), and that none of the cues would always pay $1 or $0. Fourth, two different block length conditions were included, instead of just one. The short block included 25 trials, and the long block included 75 trials. All subjects played two rounds of each block length condition, for a total of four blocks (in the order short, long, short, long) and 200 trials. Different stimuli were used in each condition and block. Different cue sets were used for each block of trials. Learning performance was assessed by computing the overall percentage of choices allocated to either of the two highest-probability cues (60 %, 70 %) in each round. All subjects first played a practice version of the learning task. They received a fixed compensation of $20 per hour, as well as 10 % of their total earnings during the task. Two additional subjects (older adults not included in the numbers reported above) were excluded from all analyses for adopting a win–stay, lose–shift strategy on every single trial.

### Results

**Fig. 5:**
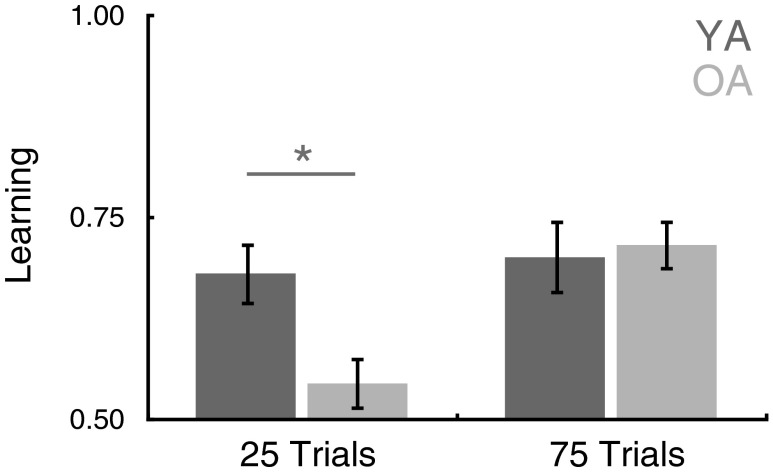
Study 2 expanded MIL task behavior. Although younger adults outperformed older adults in the short block condition (25 trials), the older adults performed as well as the younger adults in the long block condition (75 trials). Error bars indicate *SEM*s. YA = younger adults; OA = older adults. ^*^ *p* < .05

We gave participants two versions of the extended MIL task. A short condition included 25 trials to learn the stimulus–reward associations, and a long condition included 75 trials. The short condition was similar in length to the task used in Study 1 (which had included 24 trials), but an increased choice set size and reduced distance between expected values (10 % instead of 33 %) of the choice options increased the difficulty. Analysis of the behavioral results revealed a significant main effect of task length, *F*(1, 46) = 7.49, *p* < .01, such that individuals performed better on the longer blocks (75 trials) when more time was available to learn than in the shorter blocks (25 trials). The main effect of age was not significant, *F*(1, 46) = 2.30, *p* = .14, but a Block Length × Age Group interaction, *F*(1, 46) = 4.60, *p* < .05, revealed that the influence of age on learning differed between the block length conditions. Follow-up *t* tests revealed significantly higher levels of performance in the long than in the short block for the older adults, *t*(29) = 3.99, *p* < .001, but no difference between conditions for the younger adults, *t*(17) = 0.375, *p* = .71. As a result, in this larger behavioral sample, younger adults outperformed older adults in the short block condition, *t*(46) = 2.60, *p* < .05, but not in the long block condition, *t*(46) = –0.28, *p* = .78 (Fig. 5).

## Discussion

In spite of decreases across adulthood in a number of fluid cognitive abilities, researchers have found evidence of stability, and even improvement, in motivation and crystallized cognitive abilities across adulthood (Agarwal, Driscoll, Gabaix, & Laibson, 2009; Carstensen, 2006; Grady, 2008; Li, Baldassi, Johnson, & Weber, 2013; Nashiro, Sakaki, & Mather, 2012; Samanez-Larkin, 2013; Samanez-Larkin & Carstensen, 2011). The present findings are consistent with this dissociation between cognition and motivation, providing evidence for both decline and preservation of function with age. We observed both age differences in frontostriatal function related to reward learning and preserved responses to reward outcomes.

In the first study, neural correlates of prediction error were reduced during learning in older subjects, despite evidence for intact responses to reward outcomes in a task that did not involve learning. These seemingly discrepant findings are nonetheless consistent with the behavioral literature showing compromised decision making in older individuals when tasks involve novel learning—in both humans (Mata et al., 2011) and rodents (Gilbert et al., 2011; Simon et al., 2010). Although these behavioral findings may implicate regionally selective neural decline, initial human neuroimaging studies suggested that this dissociation may not be as simple as differential degradation of distinct brain regions. Instead, although individual brain regions may remain functional in some circumstances, neural circuits may not effectively transmit signals in others. Accumulating evidence suggests that reward processing recruits frontal and striatal activity independent of age (Cox et al., 2008; Hosseini et al., 2010; Samanez-Larkin et al., 2007), but that frontostriatal circuits are not as readily deployed when new learning is necessary (Aizenstein et al., 2006; Eppinger et al., 2013; Mell et al., 2009; Samanez-Larkin et al., 2010). The findings are also relatively consistent with a seminal study on reward processing and aging that showed age-related reductions in reward prediction signals, but similar if not enhanced reward outcome signals in old age (Schott et al., 2007; see also Samanez-Larkin & Knutson, 2014, for more detailed discussion of this early study).

The documented age differences in neural activity in the frontal cortex and striatum during the learning-based task are consistent with other recent evidence for abnormal prediction error signaling in old age (Chowdhury et al., 2013; Eppinger et al., 2013). However, the present study extended this recent work by directly comparing age differences in neural signals during reward tasks that both did and did not require learning (Study 1) and testing the limits of learning ability across age (Study 2).

Consistent with previous findings, younger and older adults differed in their striatal neural responses during learning. However, the lack of differences in striatal responses during the reward task that did not require learning suggested that this region may not be the core source of impairment. Instead, age differences in frontostriatal activation may result from decreased communication between a network of regions across adulthood. These age differences in connectivity could be due to a variety of disruptions, including midbrain dopamine projections to the ventral striatum and prefrontal cortex (Chowdhury et al., 2013), medial prefrontal glutamatergic input to the ventral striatum (Samanez-Larkin et al., 2012), thalamic connectivity to these regions, or some combination of these. The present findings, together with other recent work (Chowdhury et al., 2013), may identify a potential functional consequence of reduced structural connectivity. If prediction errors are not communicated effectively through the system, striatal activity may be altered in old age.

The present analyses focused on adult age differences in neural activity for monetary gain outcomes. Although a behavioral meta-analysis revealed a lack of valence bias (gain vs. loss) in decision making that does not require learning (Mata et al., 2011), some have suggested that age and valence may interact in learning (Eppinger et al., 2011, 2013). Prior studies that have included neural measures of reward sensitivity and behavioral measures of learning have provided evidence for a valence asymmetry in the neural correlates of reward anticipation (i.e., greater activation for potential gains than losses in old age) but lack of a valence asymmetry in the neural correlates of reward outcome processing and behavioral learning (Samanez-Larkin et al., 2007). Consistent with these neural findings, the present learning results did not reveal an Age × Valence interaction in learning, even in a larger behavioral sample (see Supplementary Information S4). This is inconsistent with a similar recent study that provided evidence for larger behavioral differences in learning from gains than from losses (Eppinger et al., 2013). Given inconsistencies in the behavioral literature on Age × Valence interactions in learning, in the present study we focused on the gain conditions of both tasks in order to isolate learning-related differences. However, the pattern of neural effects that emerged in the loss conditions of both tasks was similar to (but weaker than) those observed in the gain conditions. We did observe reduced MPFC activity correlated with prediction error during loss learning in older age, but the neural responses to loss outcomes in a nonlearning task did not differ between age groups (see Supplementary Information S6).

One important difference between the fMRI tasks that did and did not require learning was that different magnitudes of rewards were at stake (i.e., $0 and $1 in the MIL task, and $0, $0.50, and $5 in the MID task). A related potential limitation of Study 1 was that participants completed the MID task before the MIL task, so the reward magnitudes offered in the MIL task may have seemed smaller, and this may have contributed to reduced motivation. More specifically, an alternative account of age differences in neural response to MIL versus MID outcomes might be that older adults are less sensitive to smaller-magnitude rewards. However, the same pattern of relatively intact representation of reward value at outcome across age groups was present within the low-magnitude $0.50 trials in the MID task, which were half the magnitude of the outcomes in the MIL task (see Supplementary Information S7). Thus, the neural activation differences between tasks are not simply attributable to differences in the reward magnitudes. Another minor difference between the tasks was that the whole-brain MIL task results emerged from a parametric model of prediction error based on the fit of a reinforcement-learning model to choice data, whereas the MID task results were based on a simple comparison of gains to nongains (which does not depend on assumptions about subject choice behavior). However, a second whole-brain analysis that directly compared gains (positive prediction error) to nongains (negative prediction error) in the MIL task revealed similar results (see Supplementary Information S5). Thus, the age differences reported do not depend on the fit of the learning model.

Although we found age differences in neural activity during reward learning, older adults are not always impaired when decision making requires novel learning. In some situations older adults can even outperform younger adults (Worthy, Gorlick, Pacheco, Schnyer, & Maddox, 2011). Older adults are sometimes more likely than younger adults to adopt simpler decision strategies (Mata & Nunes, 2010; Mata, von Helversen, & Rieskamp, 2010; Mata, Schooler, & Rieskamp, 2007; Worthy & Maddox, 2012), which may facilitate some decisions. Some evidence emerged in the present findings that older adults were relatively more likely to implement simple strategies (e.g., lose–shift; see Supplementary Information S8). These findings do not clarify, however, whether strategy differences can account for age differences in neural recruitment, or whether reduced neural recruitment forces adoption of simpler strategies. Although we observed no significant interactions of age with strategy use in Study 1, the use of different strategies was more evident in Study 2. One possible interpretation is that diminished neural representation of prediction error may prevent older subjects from maintaining a strong preference for the higher-probability cue. Importantly, reduced reliance on prediction error and a greater tendency to use simple strategies may work well in some situations (Worthy et al., 2011; Worthy & Maddox, 2012). In general, the success of simple choice strategies depends on both the choice context and older peoples’ ability to take advantage of intact cognitive strengths (Mata et al., 2012).

Although we observed behavioral age differences in learning in a larger sample in Study 1 (see Supplementary Information S4) and between younger and older adults in Study 2, learning differences in the Study 1 subsample that underwent functional imaging were not significant. This was most likely due to reduced power to detect behavioral effects in this smaller group. Other studies with larger sample sizes have shown significant age differences in learning using similar tasks (Eppinger et al., 2011). Although the scanned older adults may have had less learning impairment than their peers, if this were the case, the evidence for age differences in neural activity during learning is even more striking.

The lack of age differences in the behavior of scanned subjects may also relate to specific task design details. With only two choices, subjects will sometimes choose the better option even when they have formed relatively weak preferences. To address this possibility, we conducted a separate behavioral study to explore the limits of older adults’ learning ability. Study 2 demonstrated that when task difficulty increases (by increasing choice set size and decreasing expected value differences between the cues), the learning impairment in older adults grows more pronounced. However, a task length manipulation in Study 2 also showed that, given more time to learn, older adults can approximate the levels of performance shown by younger adults even in a more difficult task.

Overall, the results suggest that neural representation of prediction error, but not reward outcome, is reduced in old age. The findings reveal a dissociation between cognition and motivation with age and identify a potential mechanism for explaining changes in learning-dependent decision making in old adulthood. In spite of the reductions in neural activity correlated with prediction error at older ages that we observed here in Study 1 and the deficits in time-limited learning in the behavioral literature, Study 2 demonstrated that supportive task conditions can remediate these deficits. Thus, in addition to identifying reductions in neurobiological function, these findings may help inform the design of interventions that will support better decisions in individuals of all ages. One implication, consistent with prior research (Mata et al., 2011), is that learning demands should be minimized for older adults who are making decisions in a novel setting.

## Electronic supplementary material

Below is the link to the electronic supplementary material.

ESM 1 (PDF 648 kb)
