# Supplementary material for: Adult age differences in frontostriatal representation of prediction error but not reward outcome
Source: Cogn Affect Behav Neurosci. 2014 May 23;14(2):672–82. doi: 10.3758/s13415-014-0297-4 (PMC4072917; doi:10.3758/s13415-014-0297-4)

Adult age differences in frontostriatal representation of prediction error but not reward outcome

Samanez-Larkin, Worthy, Mata, McClure, Knutson

2014

Supplementary Information

S1: Description of Study 1 and Study 2 Samples

S2: fMRI Task Schematics

S3: MIL Task PE Whole-Brain Regression Model

S4: MIL Task Performance in Larger Behavioral Sample

S5: Additional MIL Task Whole-Brain fMRI Results

S6: Loss Condition Results

S7: MID Task Low Magnitude Timecourses

S8: Model Comparisons for Studies 1 and 2

S1: Description of Study 1 and Study 2 Samples

	**Study 1 (fMRI sample)**	**Study 1 (fMRI sample + behavior only subsample)**	**Study 2 (behavior only)**
N	39	77	Y: 18<br>O: 30
Age range	22–85	20–85	Y: 19–33<br>O: 67–86
% female	54%	52%	52%
Reward tasks completed	MIL (N=39)<br>24 trials/condition<br>MID (N=37)	MIL (N=77)<br>12 trials/condition	modified MIL (N=48)

Note that different groups of subjects participated in Studies 1 and 2.

S2: fMRI Task Schematics

In the Monetary Incentive Learning (MIL) task, subjects view each cue (2 s each), select one when presented with the word "Choose" (max 3.5 s), view their highlighted choice (4 s – choice reaction time), view the monetary outcome of their choice (2 s), and then fixate centrally for a variable inter-trial interval (2–6 s). In the Monetary Incentive Delay (MID) task, subjects view an explicit cue which indicates the trial type and reward magnitude (2 s), fixate centrally for a variable delay (2 – 2.5 s), respond as quickly as possible to a target, fixate centrally (4 s – (pre-target delay + choice RT)), view performance feedback (Hit!, Miss!) and the monetary outcome of their choice (2 s), and then fixate centrally for a variable inter-trial interval (2–6 s).

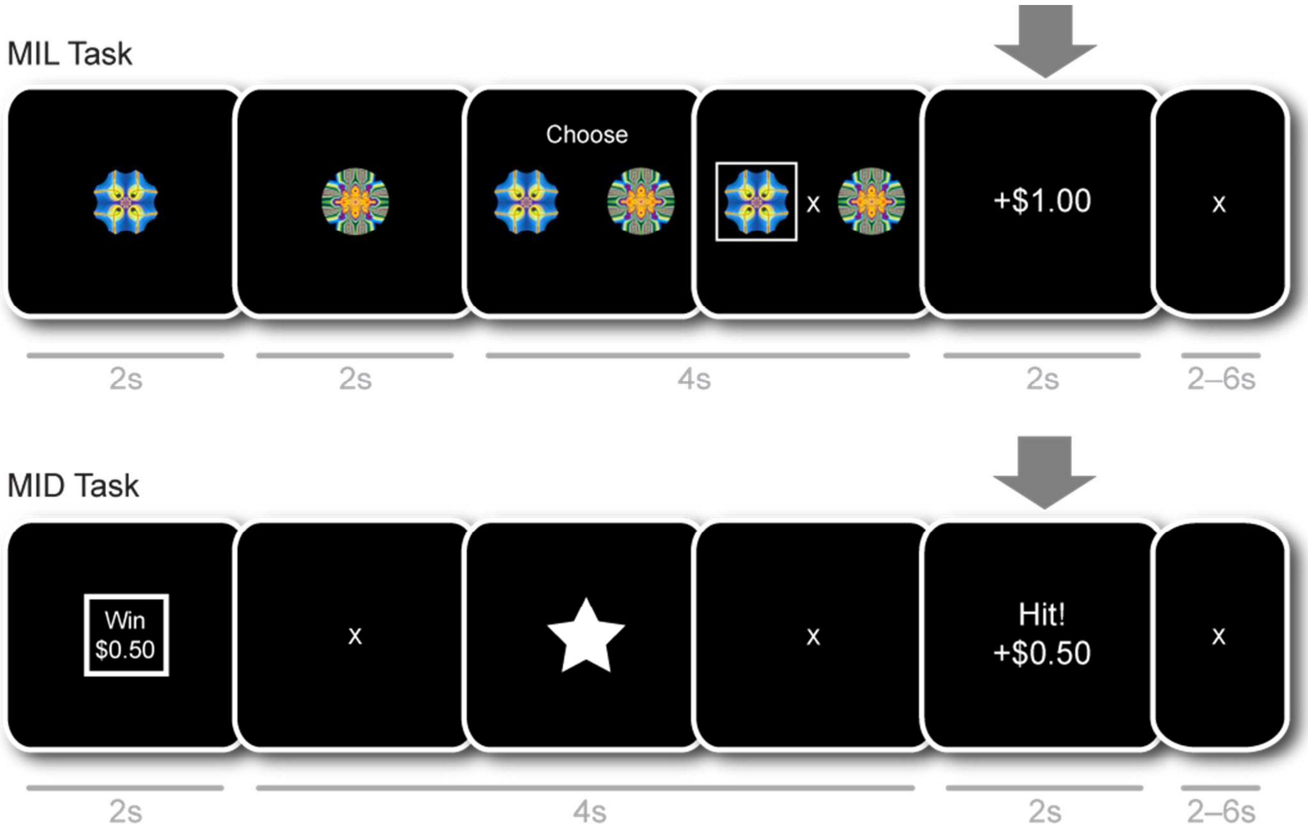

S3: MIL Task PE Whole-Brain Regression Model

The prediction error regression model used computationally-derived estimates of value produced by a standard reinforcement learning model fit to individual subject choices (O'Doherty, et al., 2003; Sutton & Barto, 1998). Expected values (*V*) were computed to represent the reward expected by selecting each cue. The values of *V* for each cue were initialized at 0 and updated for the chosen cue on each trial according to

$$V(s_i) \leftarrow V(s_i) + \alpha[r_i - V(s_i)] \quad (1)$$

where s_i is the cue encountered on trial *i* and α is the learning rate. Learning is mediated by a prediction error (the bracketed portion of Equation 1) between the reward received (r_i) and the expected value of the chosen option $V(s_i)$. The prediction error is positive if the reward received is larger than expected and negative if the reward received is smaller than expected. Prediction errors were modeled at outcome in the MIL task (but reward predictions, *V*, were not included in the model). Learning is modulated by a learning rate, or recency parameter (α; $0 < \alpha < 1$), that determines the degree to which subjects update the reward expectations based on the most recently received rewards. As this rate approaches 1 greater weight is given to the most recent reward. To fit behavior, the probability of selecting each action was assumed to follow a softmax rule, which asserts that the probability of selecting a cue based on the expected values:

$$P(s_i) = \frac{e^{[\beta \bullet V(s_i)]}}{\sum_{j=1}^{n} e^{[\beta \bullet V(s_j)]}} \quad (2)$$

Here β is a decision slope parameter that determines the degree to which the option with the highest EV is chosen. As β becomes larger the highest valued option is chosen more often, and as β approaches 0 all options are chosen equally often.

The constants α (learning rate) and β (decision slope) were adjusted to maximize the probability of the observed choices under the model. Best-fitting values for gain learning were $\alpha = 0.34$ (95% CI: 0.25–0.43) and β = 0.12 (95% CI: 0.06–0.17), and for loss learning were $\alpha = 0.39$ (95% CI: 0.27–0.51) and β = 0.22 (95% CI: 0.16–0.28). Learning rates were not correlated with task performance in either the gain condition, β = .21, p = .20, or loss condition, β = .25, p = .12. Decision slopes were positively correlated with task performance in the gain condition, β = .50, p < .01, but not the loss condition, β = .23, p = .14. Despite these inconsistent associations between model parameters and overall task performance, the predicted probabilities of choosing the higher probability cue on each trial over time provided a relatively accurate fit to the choices of the subjects (see figure below). For fMRI analyses, the learning model was used to create regressors with a single set of parameters (mean) across all subjects, since the use of individual subject fits yielded less consistent results. Continuous representations of prediction errors ($r_i - V(s_i)$ at outcome were used in the fMRI regression model.

Supplementary Figure 1. Reinforcement learning model fit. Line is prediction based on model parameters. Dots are average percentage of choices for each cue over 24 trials in the MIL task.

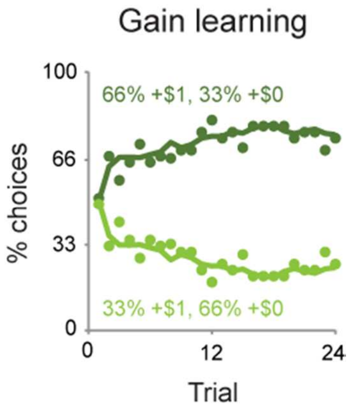

S4: MIL Task Performance in Larger Behavioral Sample

Seventy-seven adults (age mean = 55, SD = 17, range 20–85) played 12 trials per condition (gain, loss, neutral). Half of the sample (39 adults) were the same individuals who completed the fMRI version of the task. For these analyses, only the first half of their trials (12 trials per condition) were included in the measures of learning performance (to equate trial numbers and learning phase). The additional thirty-eight subjects in the sample were recruited using the same market research firm, but did not complete the 24 trial version of the MIL task while undergoing fMRI. They only played the first half of the learning task (12 trials per condition). Separate analyses of this dataset are reported in a recent publication that focuses on long-term financial outcomes in life (i.e., asset and debt accumulation) rather than age differences in learning ability (Knutson et al., 2011). All subjects in this sample of 77 were recruited from the community in exactly the same way. They were all initially invited to participate in both neuroimaging and behavioral phases of experiments in the lab, although some of them were either ineligible for imaging or opted to only complete a behavioral session.

Analysis in this larger behavioral dataset revealed a main effect of age, $F_{1,75} = 6.57$, $p < .05$, such that learning performance was higher in younger compared to older adults. There was a non-significant main effect of task condition (gain, loss), $F_{1,75} = 0.03$, $p = .86$, and a non-significant interaction of age and task condition (gain, loss) $F_{1,75} = 0.21$, $p = .65$. In a separate model that included both a linear and quadratic effect of age, the main effect of age was still significant, $p = .01$, but the quadratic effect of age was non-significant, $p = .45$. In summary, older adults performed more poorly across both conditions of the learning task in this larger behavioral sample focused on the early stages of learning.

Learning performance scores by age and task condition are plotted below in Supplementary Figure 2.

Supplementary Figure 2. MIL task learning performance in larger behavioral sample (12 trials per condition). Top row depicts average percentage of choices of the high probability option averaged across the gain and loss learning conditions by age (upper left) and by age group (upper right). Bottom row depicts average percentage of choices of the high probability option for the gain and loss learning conditions by age (lower left) and by age group (lower right). Error bars are s.e.m.

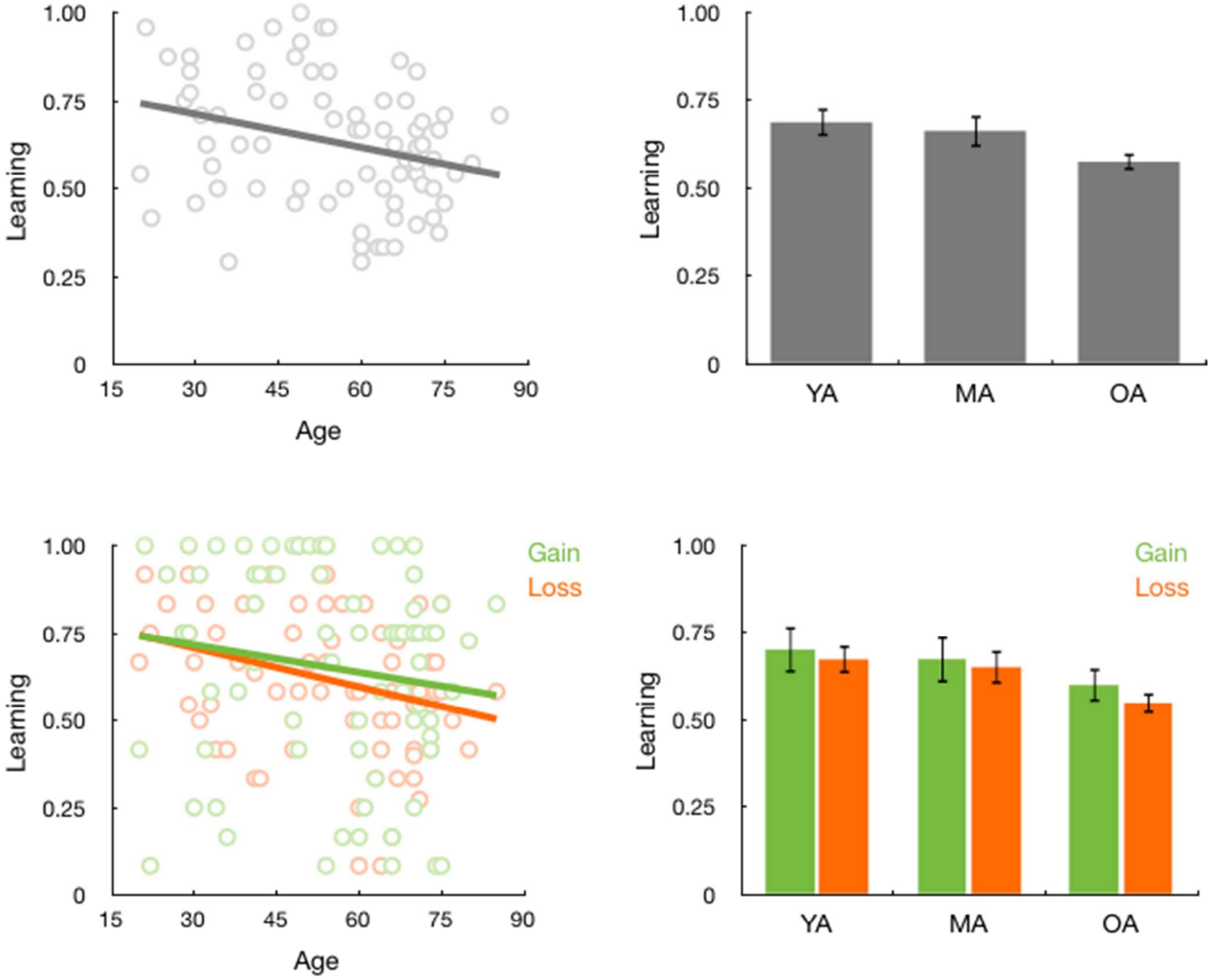

S5: Additional MIL Task Whole-Brain fMRI Results

In a second and separate model (that is more analogous to the MID task reward outcome model), we conducted a direct contrast of positive PE versus negative PE. This additional “PE-collapsed” MIL task regression model consisted of a set of two orthogonal regressors of interest: all positive PE’s (+$1) versus all negative PE’s (+$0) on gain trials, all positive PE’s (–$0) versus all negative PE’s (–$1) on loss trials. Additional regressors of non-interest included residual motion and baseline, linear and quadratic trends. Not surprisingly, similar results emerge in the gain condition. Across age, medial prefrontal and ventral striatal regions show greater activation for positive compared to negative PEs in the gain condition, and the difference between the functional representation of positive compared to negative PEs is reduced with age in the anterior cingulate and ventral striatum.

Supplementary Table 1. Prediction error at outcome (PPE vs NPE) in task that required learning (MIL). P < .001. N = 39

**Region**	**R**	**A**	**S**	**Z**	**Voxels**	**Volume (mm^3)**
Across all subjects						
R Medial Frontal Gyrus	4	49	–3	4.12	23	1213
R Ventral Putamen	19	8	–7	4.84	6 [SVC]	316
L Inferior Parietal Lobule	–52	–52	46	4.02	7	369
R Cuneus	8	–71	19	3.70	8	422
Linear effect of age (OA > YA)						
L Anterior Cingulate	–4	38	8	–4.20	9	475
R Lentiform Nucleus / Nucleus Accumbens	8	0	–3	–4.37	13	686
L Lingual Gyrus / Posterior Cingulate	–11	–52	4	–4.02	12	633
Quadratic effect of age						
Anterior Cingulate	0	38	4	–4.20	12	633

Supplementary Figure 3. Alternate MIL task whole brain model results (PPE vs NPE). (A) Regions that showed a significant difference in activation between PPE and NPE at outcome across age. (B) Regions of the brain that showed age differences in activation between PPE and NPE at outcome. Blue corresponds to negative z-scores which indicate a reduced difference between PPE and NPE as age increased. R = right. R/L, A/P, or S/I value listed in upper corner of each statistical map. Anatomical underlay is an average of all subjects' spatially normalized structural scans.

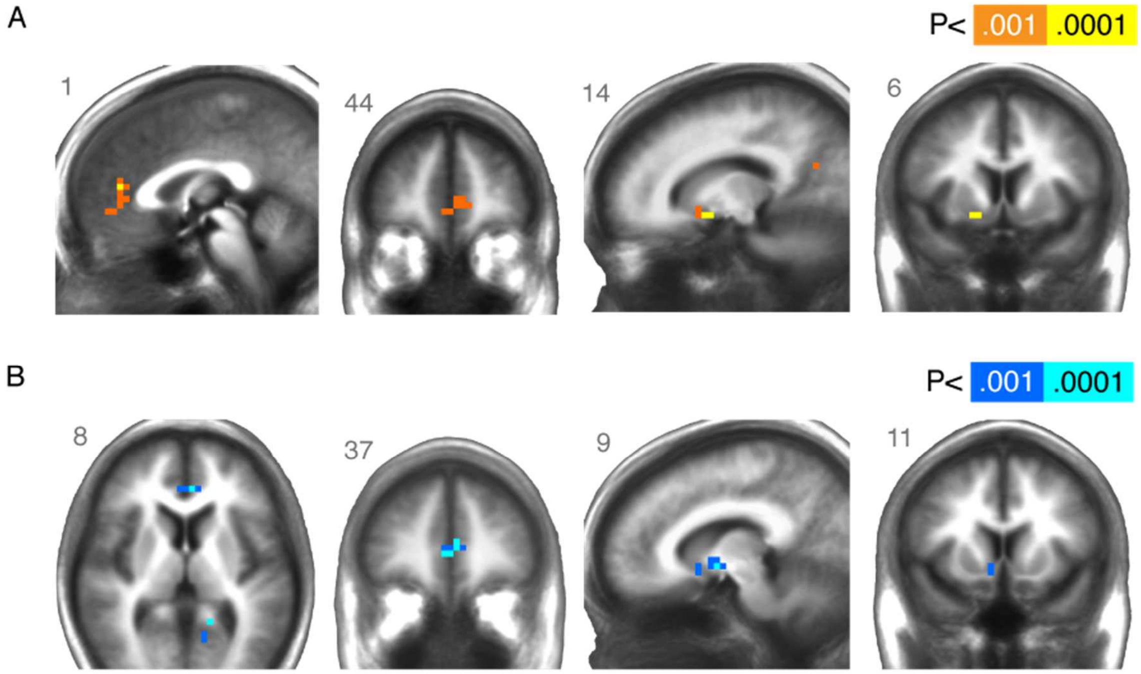

S6: Loss Condition Results

MIL task. For the task that required learning, there were no regions that showed a significant difference in activation between loss outcomes (PPE vs NPE) across subjects at the cluster-corrected threshold (see Supplementary Table 2). Reducing the cluster-correction revealed a five-voxel subthreshold cluster in the right anterior cingulate (RAS = 4, 15, 23; Z = 3.69) with significantly greater signal for loss avoidance (–$0) than actual losses (–$1) across subjects.

A linear effect of age was observed in a dorsomedial frontopolar region at the cluster-corrected whole-brain threshold (see Supplementary Table 2 and Supplementary Figure 5 middle). Reducing the cluster-correction revealed a five-voxel subthreshold cluster in the right parahippocampal gyrus / amygdala where the difference between PPE and NPE was smaller with age (RAS = 23, –15, –11; Z = –3.77).

Supplementary Table 2. Prediction error at outcome (PPE vs NPE) on loss learning trials in task that did require learning (MIL). P < .001. N = 39

**Region**	**R**	**A**	**S**	**Z**	**Voxels**	**Volume (mm^3)**
Across all subjects						
none						
Linear effect of age (OA > YA)						
L Medial Frontal Gyrus	0	60	19	–3.78	8	422
Quadratic effect of age						
none						

MID task. For the task that did not require learning, a region of the ventral putamen showed greater activation for loss avoidance (–$0) compared to losses (–$0.50, –$5.00) across subjects (see

Supplementary Table 3 and Supplementary Figure 6). However, there were no regions that showed significant age differences for this contrast.

Supplementary Table 3. Loss avoidance at outcome (–0 vs –$) in task that did not require learning (MID). P < .001. N = 37

**Region**	**R**	**A**	**S**	**Z**	**Voxels**	**Volume (mm^3)**
Across all subjects						
R Ventral Putamen	23	8	–3	3.93	7	369
Linear effect of age (OA > YA)						
none						
Quadratic effect of age						
none						

S7: MID Task Low Magnitude Timecourses

One important difference between the MID and MIL task was that different magnitudes of rewards were at stake. The MIL task included two reward levels, $0, $1, and the MID task included three reward levels, $0, $0.50, $5. An alternative account of the presence of age differences in the MIL task and absence of age differences in the MID task is that older adults are less sensitive to lower magnitude rewards. To test this possibility we plotted timecourses for the low magnitude ($0.50) condition from the MID task for each region of interest. The same pattern of results emerges ruling out this alternative account. Older adults show signal change differences between $0.50 gains and $0 nongains in the MID task. Thus, the differences between tasks are not simply due to differences in reward magnitudes.

Supplementary Figure 4. Black lines are +$0.50 outcomes (successfully hit target) and grey lines are +$0.00 outcomes (missed the opportunity to win $0.50).

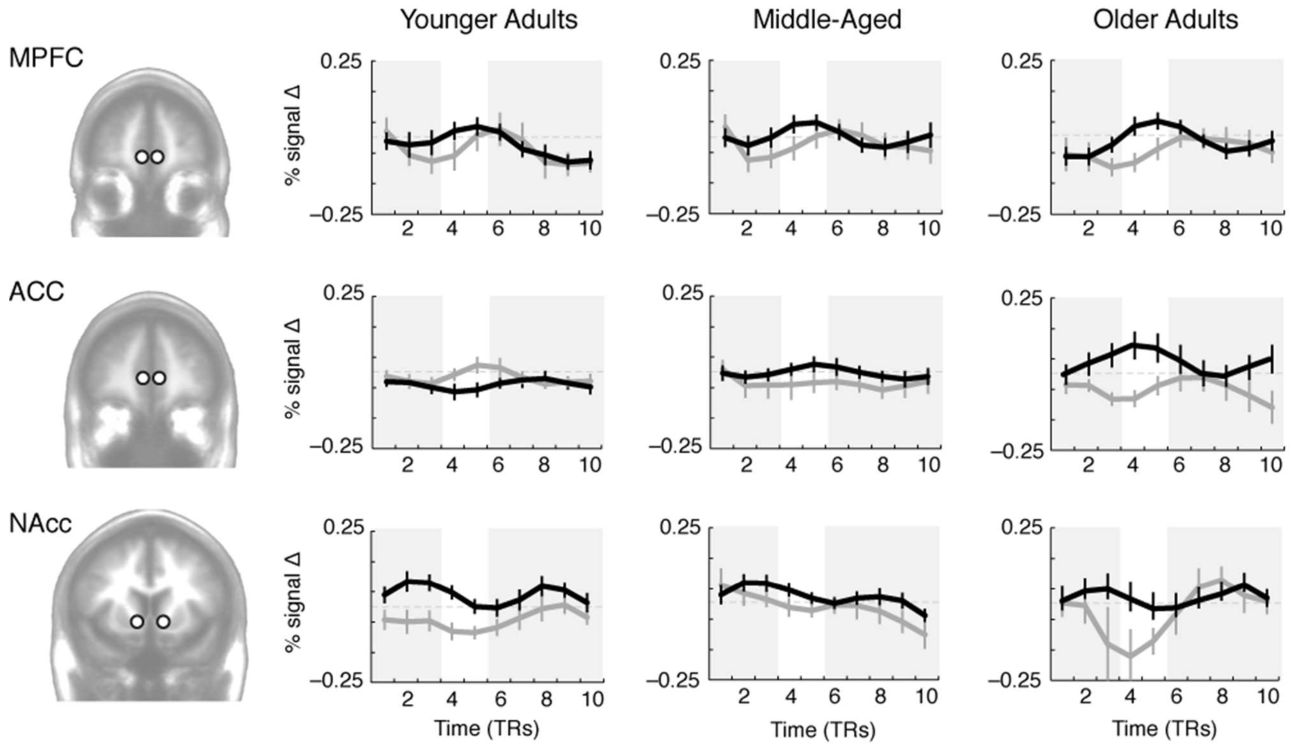

S8: Model Comparisons for Studies 1 and 2

Additional model fitting explored whether adults in either study were better fit by a Rescorla-Wagner reinforcement learning (RL) or win-stay/lose-shift (WSLS) model. Additionally, we independently examined win-stay and lose-shift across age groups and tasks. For all analyses and discussion below both gains (+$1) in the gain condition and non-losses (–$0) in the loss condition will be categorized as “wins” for the estimation of win-stay. Likewise, both losses (–$1) in the loss condition and non-gains (+$0) in the gain condition will be categorized as “losses” for the estimation of lose-shift.

In Study 1, a marginally significant main effect of task condition, $F_{1, 36} = 3.98$, $p = .05$, suggested that subjects were slightly better fit by RL than WSLS in the gain relative to the loss condition. Within the two valence conditions, subjects across age were better fit by RL than WSLS in the gain condition, $t_{38} = 2.07$, $p < .05$, but there was not a significant difference between the fit of RL and WSLS in the loss condition, $t_{38} =1.35$, $p = .19$. There was a non-significant main effect of age group, F2, 36 = 2.42, p = .10, and non-significant condition by age group interaction, F2, 36 = 0.19, p = .82. See Supplementary Figure 5A.

A separate ANOVA on the two parameters from the WSLS model (win-stay, lose-shift) revealed a main effect of task condition, $F_{1, 36} = 11.17$, $p < .005$, such that both behaviors (win-stay and lose-shift) were higher in the loss compared to gain condition across age groups. A main effect of WSLS, $F_{1, 36} = 138$, $p < .0001$, revealed much higher parameter values for win-stay than lose-shift across task valence conditions and age groups. A significant WSLS by age group interaction, $F_{2, 36} = 4.53$, $p < .05$, suggested that younger adults were more likely to win-stay than lose-shift compared to the older adults. However, follow-up tests revealed non-significant effects of age for win-stay on gain trials, p = .21, win-stay on loss trials, p = .08, lose-shift on gain trials, p = .13, and lose-shift on loss trials, p = .28. The condition (gain, loss) by WSLS (win-stay, lose-shift) by age group interaction was not significant, $F_{2, 36} = 0.64$, p = 54. See Supplementary Figure 5B.

In Study 2, there was a non-significant main effect of task condition, $F_{1, 48} = 0.06$, $p = .81$, a non-significant main effect of age group, $F_{1, 48} = 2.05$, $p = .16$, and a non-significant age by condition interaction, $F_{1, 48} = 1.37$, $p = .25$. However, across subjects WSLS provided a better fit than RL for the short condition, $t_{49} = 2.77$, $p < .01$, and WSLS and RL provided an equally good fit for the long condition, $t_{49} = 0.74$, $p = 46$. See Supplementary Figure 5C.

A separate ANOVA on the two parameters from the WSLS model (win-stay, lose-shift) revealed a non-significant main effect of task condition, $F_{1, 48} = 1.27$, $p = .27$, such that both behaviors (win-stay and lose-shift) were equivalent in the short and long conditions across age groups. A main effect of WSLS, $F_{1, 48} = 6.77$, $p < .05$, revealed higher parameter values for win-stay than lose-shift across task length conditions and age groups. A significant WSLS by age group interaction, $F_{1, 48} = 14.10$, $p < .001$, suggested that younger adults were more likely to win-stay than lose-shift compared to the older adults. Follow-up tests revealed non-significant effects of age for win-stay in the short condition, $p = .16$, and win-stay in the long condition, $p = .25$, but significant age differences in lose-shift in the short condition, $p < .001$, and lose-shift in the long condition, $p < .0001$. The condition (short, long) by WSLS (win-stay, lose-shift) by age group interaction was not significant, $F_{1, 48} = 0.82$, $p = .37$. See Supplementary Figure 5D.

Supplementary Figure 5. (A; upper left) Fit of WSLS model relative to RL model in Study 1. Y-axis is difference in log-likelihood (RL – WSLS). Both models have two parameters so their log likelihoods can be compared directly. (B; upper right) Average best-fitting win-stay and lose-shift parameters (y-axis) across age groups and task conditions (x-axis) in Study 1. (C; lower left) Fit of WSLS model relative to the RL model in Study 2. Y-axis is difference in log-likelihood (RL – WSLS). Both models have two parameters so their log likelihoods can be compared directly. (D; lower right) Average best-fitting win-stay and lose-shift parameters (y-axis) across age groups and task conditions (x-axis) in Study 2. Error bars are S.E.M.

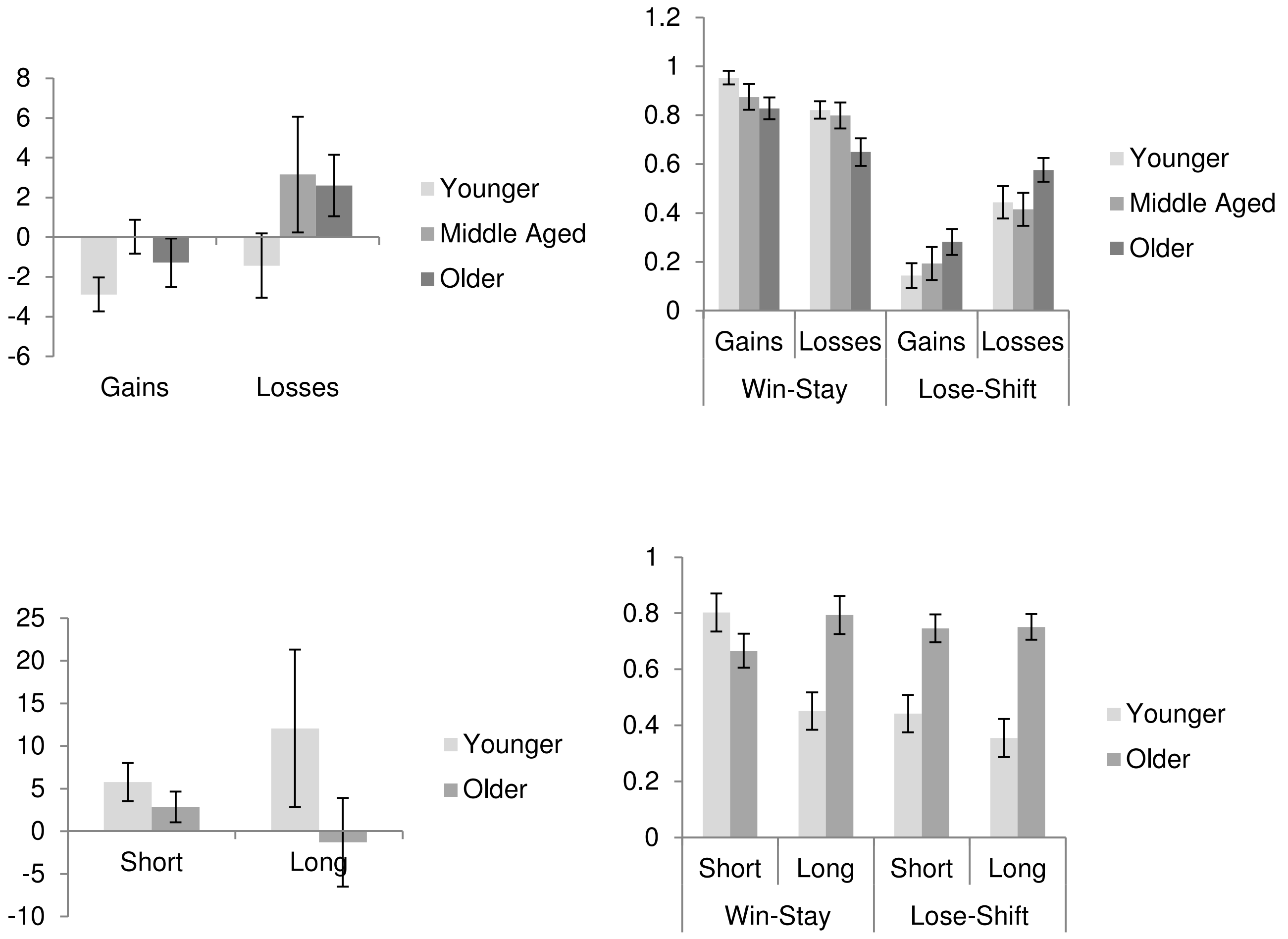

Supplement: Supplementary file 1 — (PDF 648 kb) [file 13415_2014_297_MOESM1_ESM.pdf]
